# Diagnosis of non-puerperal mastitis based on “whole tongue” features: non-invasive biomarker mining and diagnostic model construction

**DOI:** 10.3389/fcimb.2025.1602883

**Published:** 2025-07-28

**Authors:** Siyuan Tu, Yulian Yin, Lina Ma, Hongfeng Chen, Meina Ye

**Affiliations:** Department of Breast Surgery, Longhua Hospital, Shanghai University of Traditional Chinese Medicine, Shanghai, China

**Keywords:** non-puerperal mastitis, tongue diagnosis, tongue microbiota, high through put sequencing, machine learning model

## Abstract

**Background:**

Non-puerperal mastitis (NPM) arises from heterogeneous factors ranging from autoimmune dysregulation to occult infections. To establish a diagnosis, biopsy is reliable but invasive. Imaging exhibits a limited specificity and may cause diagnostic delays, patient discomfort, and suboptimal management. Inspired by non-invasive tongue diagnosis in traditional Chinese medicine, this study integrated tongue-coating microbiota profiling and AI-quantified tongue image phenotyping to establish an objective, non-invasive diagnostic framework for NPM.

**Methods:**

A total of 100 NPM patients from the Breast Surgery Department of Longhua Hospital and 100 healthy volunteers were included. Their clinical characteristics, tongue images, and tongue-coating microbiota data were collected. Features of tongue images (detection, segmentation, and classification) were quantitated and extracted via deep learning. The microbiota composition was assessed using 16S rRNA gene sequencing (V3–V4 region) and bioinformatic pipelines (QIIME2, DADA2). Based on clinical, imaging, and microbial features, three machine learning models—logistic regression (LR), support vector machine (SVM), and gradient boosting decision tree (GBDT)—were trained to distinguish NPM.

**Results:**

The GBDT model achieved a superior diagnostic performance (AUROC = 0.98, accuracy = 0.95, and specificity = 0.95), outperforming the LR (AUROC = 0.98, accuracy = 0.95, and specificity = 0.90) and SVM models (AUROC = 0.87, accuracy = 0.80, and specificity = 0.75). Integration of clinical characteristics, tongue image features, and bacterial profiles (at the genus/family level) yielded the highest accuracy, whereas models using a single class of features showed a lower discriminatory ability (AUROC = 0.90–0.91). Key predictors included *Campylobacter* (12%), waist–hip ratio (11%), and *Alloprevotella* (6%).

**Conclusions:**

Integrating clinical characteristics, tongue image features, and tongue-coating microbiota profiles, the multimodal GBDT model demonstrates a high diagnostic accuracy, supporting its utility for early screening and diagnosis of NPM.

## Introduction

Non-puerperal mastitis (NPM) is an entity of inflammatory breast diseases including mammary duct ectasia, idiopathic granulomatous mastitis (IGM), periductal mastitis, and tuberculous mastitis (Kasales et al., 2014; Scott, 2022; Shi et al., 2022). While NPM is detected in only 4% to 5% of biopsies for benign breast diseases (Shi et al., 2022), its morbidity has kept rising over the last two decades, and currently, it occurs in adult women of all ages with a prolonged and recurrent course (Verghese and Ravikanth, 2012; Yuan et al., 2022). However, the etiology of NPM is still elusive, which challenges early diagnosis and subsequent treatment (Gopalakrishnan et al., 2015). Due to its heterogeneous etiology (e.g., microbial infections) (Li et al., 2022; Tariq et al., 2022), autoimmune responses (Chougule et al., 2015), ambiguous clinical features (resembling invasive ductal carcinoma and inflammatory breast cancer in terms of symptoms (Chen et al., 2023), and nonspecific imaging findings (non-mass enhancement or irregular rim enhancement with blurred margins) (Fazzio et al., 2016), how to make a definite diagnosis of NPM remains a concern in clinical scenarios.

Histopathological analysis is a golden standard for diagnosing NPM (Liang et al., 2022). However, a possible misdiagnosis with malignant diseases still exists due to the complications with core needle biopsy (e.g., bleeding, sinus formation, and pain) and limited lesions taken for tests (Yuan et al., 2022). On the other hand, deep learning models are making the diagnosis of NPM more non-invasive, convenient, and inexpensive. A nomogram based on multiparametric sonogram and radiomics features (lesion diameter, orientation, echogenicity, shape and tubular extension features, and the American College of Radiology Breast Imaging Reporting and Data System score) can well differentiate IGM from invasive breast cancer (IBC) (Ma et al., 2023a); however, this model does not show a high stability due to the variation in sonographic variables among sonographers. Magnetic resonance imaging (MRI)-based whole-lesion histogram and texture analysis can be used to differentiate IGM from IBC, with a 79.9% accuracy rate, but this analysis depends on high-quality manual segmentation, different MR systems, and single-shot diffusion weighted imaging (Zhao et al., 2020). MRI can rule out malignancy with a high sensitivity, but its specificity decreases in the absence of mass enhancement (Soylu et al., 2023). Accordingly, it is urgent to explore for new non-invasive biomarkers and improve the model’s performance in the diagnosis of NPM.

Tongue-coating microbiota are involved in the progression of systemic diseases (Shapira et al., 2013), such as rheumatic immunological disorders, respiratory, circulatory, urinary, and digestive system diseases as well as dental caries and other oral ailments (Gao et al., 2018). Mechanistic studies have revealed that some differentially enriched tongue-coating microbial species can serve as disease biomarkers, providing scientific evidence supporting the value of tongue diagnosis, a method in traditional Chinese medicine (TCM). The connection between tongue microbiota and NPM is still controversial (Betal and Macneill, 2011; Le Fleche-Mateos et al., 2012; Renshaw et al., 2011). Various diseases can be defined through tongue diagnosis (Liu et al., 2023), including IGM (Chen et al., 2022), indicating the possibility of using the “whole tongue” to diagnose NPM. However, no research has analyzed the diagnostic potential of tongue-coating microbiota for NPM.

**Figure 1:**
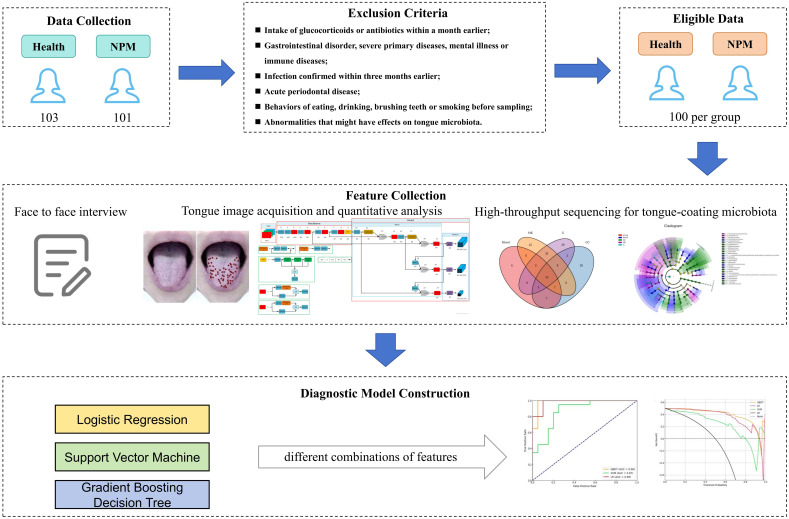
Overall flow of this study.

Here we created a gradient boosting decision tree (GBDT) model, which encompassed significant clinical characteristics, “whole tongue” imaging, and microbiota features, and evaluated its clinical value in the early screening of NPM (**Figure 1**).

## Materials and methods

### Data

From April 2021 to November 2023, a total of 101 NPM patients from the Breast Surgery Department of Longhua Hospital Affiliated to Shanghai University of TCM and 103 healthy volunteers were recruited. All patients were pathologically diagnosed with NPM by needle biopsy or post-surgery histopathological analysis. Healthy control participants presented no clinically diagnosed diseases and were not on medications. Additionally, excluded were those with (1) intake of glucocorticoids or antibiotics within a month earlier, (2) duodenal ulcer, gastric ulcer, gastrorrhagia, or other gastrointestinal disorder, (3) severe primary diseases or mental illness, (4) immune diseases, such as rheumatoid arthritis, systemic lupus erythematosus, and autoimmune skin diseases, (5) infection confirmed within 3 months earlier, (6) concurrent acute periodontal disease, (7) behaviors of eating, drinking, brushing teeth, or smoking before sampling, and (8) other abnormalities that might have effects on tongue microbiota. All of the included participants received a face-to-face interview, had tongue imaging, and provided tongue coating samples. Finally, 100 participants were assigned to each group. The study protocol was approved by the Ethics Committee of Longhua Hospital Affiliated to Shanghai University of Traditional Chinese Medicine (2021LCSY047). All participants provided written informed consent.

A questionnaire survey was performed to collect clinical characteristics, including age, height, weight, waist circumference, hip circumference, systolic pressure (SP), and diastolic pressure (DP). Body mass index (BMI) was computed as weight in kilograms divided by the square of height in meters. Waist–hip ratio (WHR) was computed as waist circumference divided hip circumference.

### Tongue image acquisition and quantitative analysis

**Figure 2:**
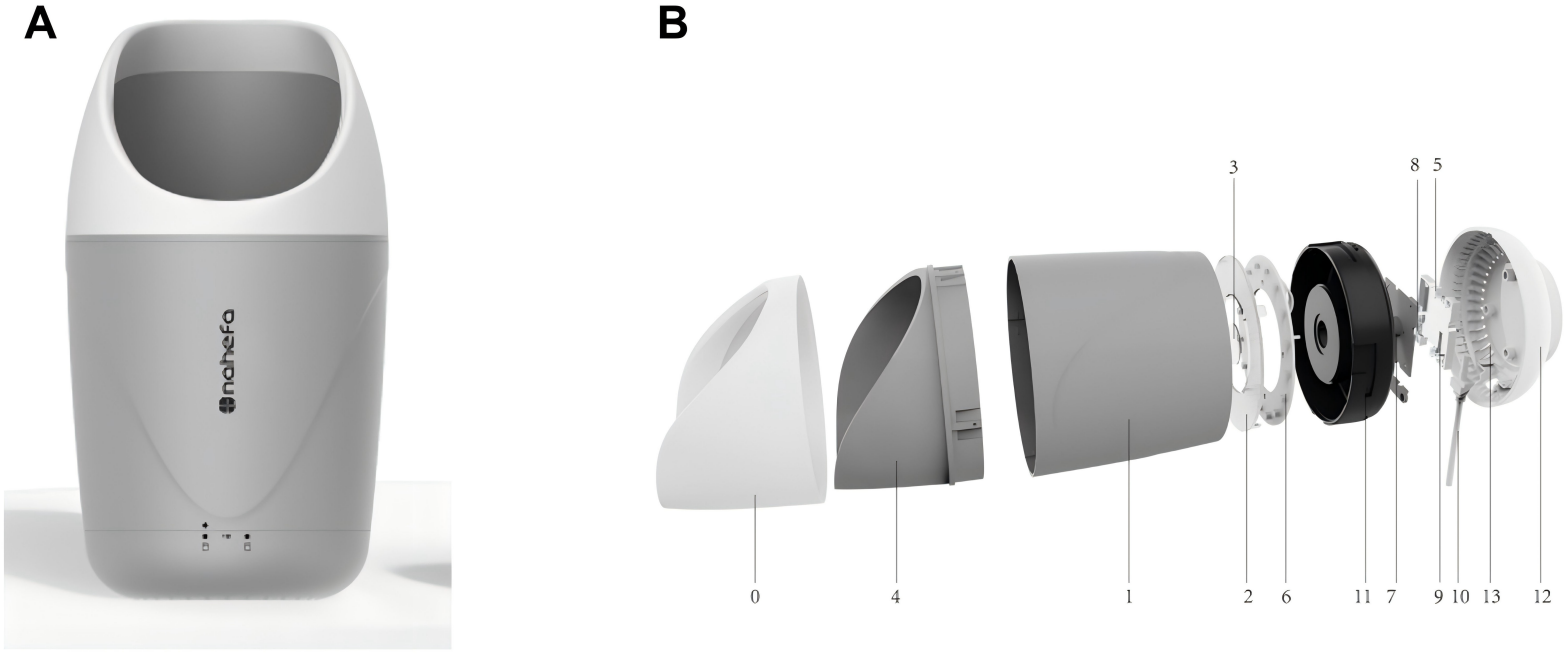
Composition of GMSX001 tongue diagnosis instrument. **(A)** Overall view. **(B)** Exploded view (1, light concentrator cylinder; 2, uniform light plate; 3, camera lenses; 4, fixed base; 5, DSP image processing chips; 6, lamp panel modules; 7, camera sensors and modules; 8, LED drivers; 9, power management chips; 10, data cable; 11, internal reinforced liner; 12, pedestal; 13, heat emission hole). DSP, digital signal processor; LED, light-emitting diode.

Before sampling tongue-coating microbiota, tongue images were collected by researchers trained on a tongue diagnosis device (GMSX001, Shanghai National Health Company, Shanghai, China) (**Figure 2**), which contains a SONY IMX179 photosensitive chip, with a closed light source, color temperature of 5,600 K, illumination of 1,200 lx, and a color rendering index greater than 85 Ra. All of the images obtained were processed into the JPG format. Each tongue was imaged at least two times. The images with nebulization, underexposure, overexposure, stained tongue coating, and abnormal tongue shape were removed.

We extracted the color and texture features of the tongue by applying Nahefa Cloud System V2.0 developed by Shanghai National Health Company. After color correction and image segmentation, the system automatically distinguished the tongue body from the tongue coating. The tongue image quantification system was constructed based on techniques of deep learning object detection (Wang, 2023), deep learning image segmentation (Chen et al., 2018), and deep learning image classification (He, 2016).

**Figure 3:**
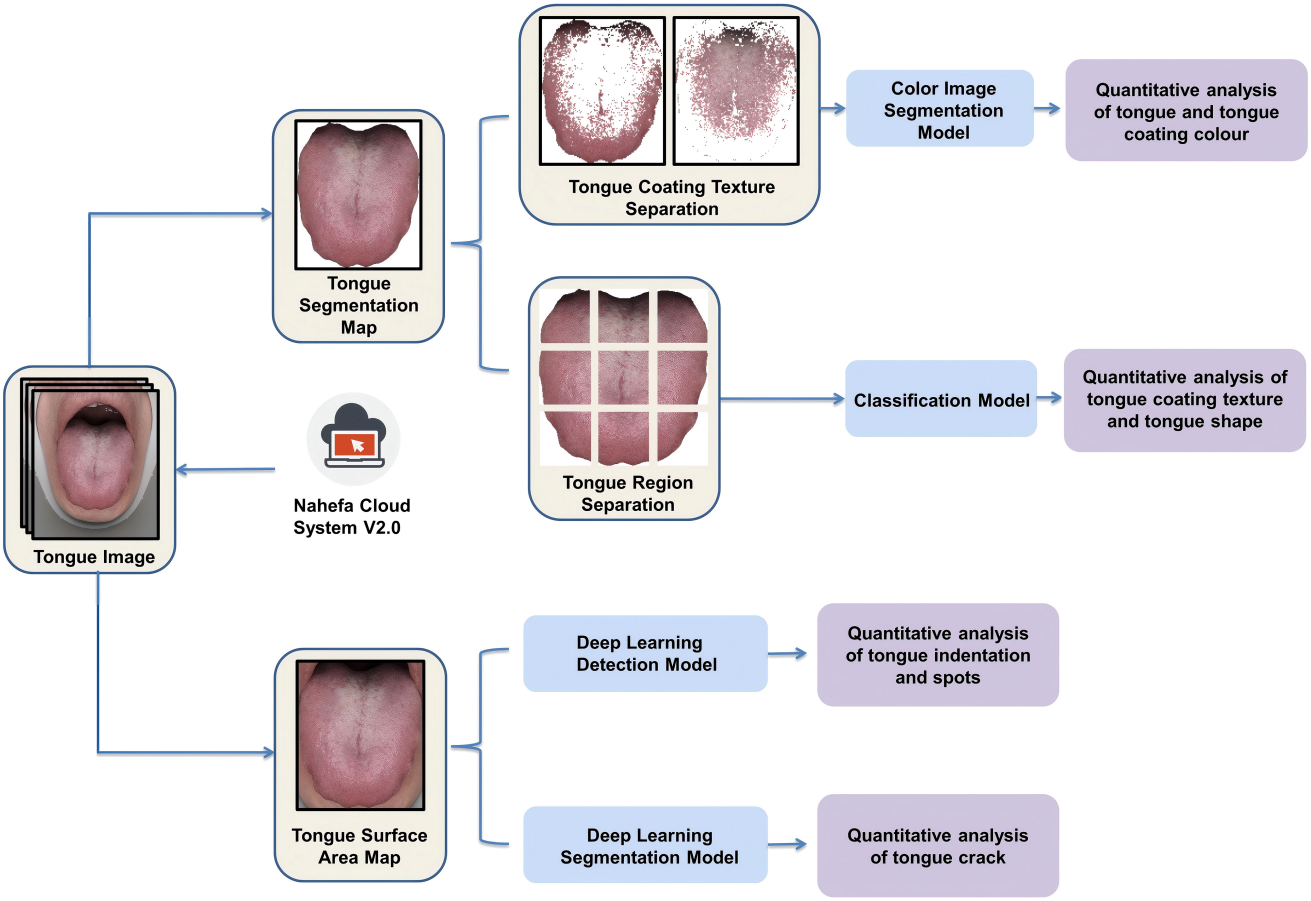
Process of extracting tongue image features from multiple dimensions.

Three attending physicians in TCM labeled the tongue features on the basis of diagnostics in Chinese medicine. After labeling, three TCM experts conducted a spot check of the labeling quality. The model was trained after the labeling was considered qualified. Each model used a different evaluation method: mAP for the detection model, acc for the classification model, and mIoU for the segmentation model. Tongue and tongue coating colors, tongue coating texture, and tongue shape were calculated by using the deep learning image classification model that had been trained through the diagnostic results of medical experts (**Figure 3**).

The tongue coating texture and tongue shape were calculated as follows:

1. The tongue surface area was divided into nine parts.
2. Each part was given a score by its own classification model.
3. The nine parts’ sum score was divided by “nine times the feature level” in order to obtain the quantitative value of the tongue features.

**Figure 4:**
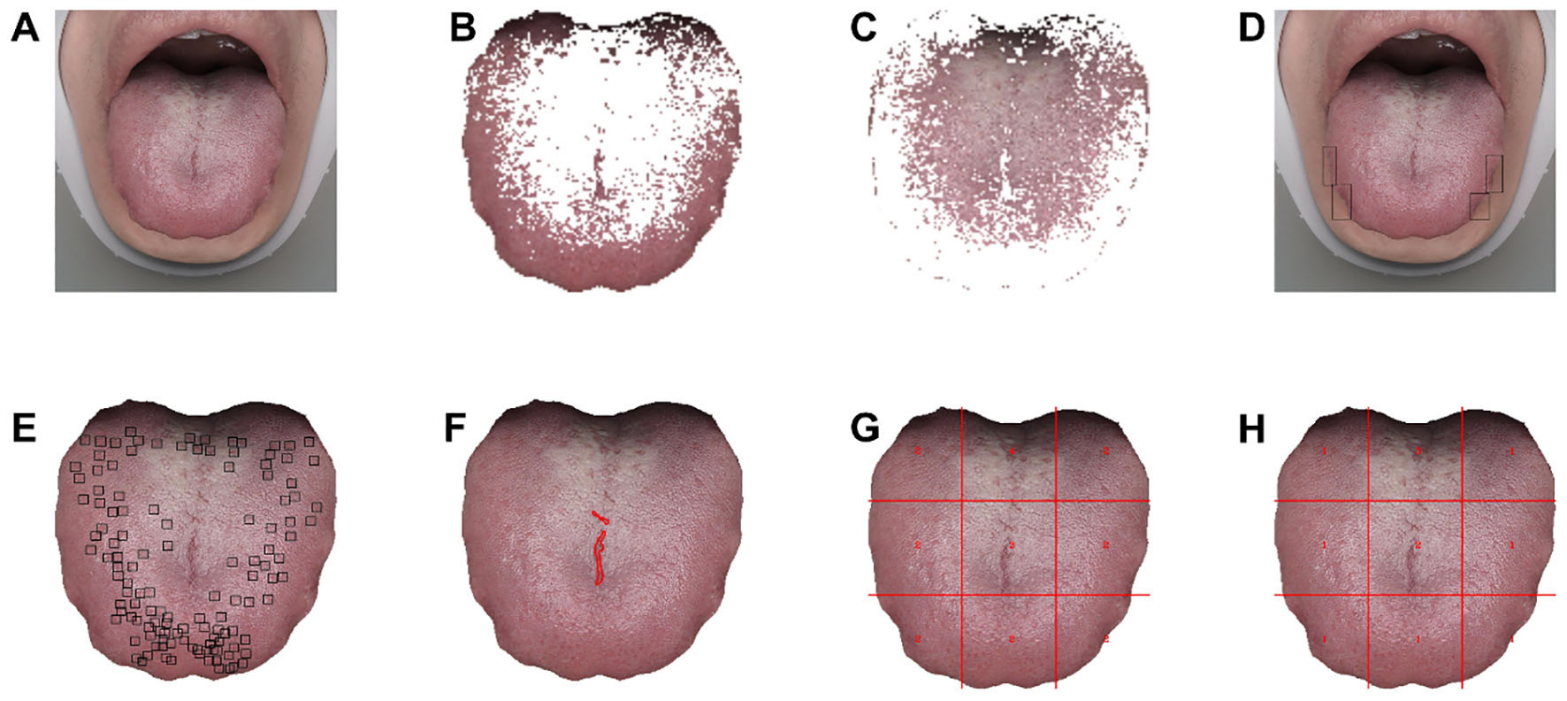
Visualized quantitative analysis based on tongue images. **(A)** Original tongue image. **(B, C)** Tongue coating texture image. **(D)** Teeth indentations on tongue image. **(E)** Tongue spots image. **(F)** Tongue cracks image. **(G)** Tongue coating thickness image (1 = no tongue coating, 2 = thin tongue coating, 3 = mild thick tongue coating, 4 = severe thick tongue coating). **(H)** Tongue coating grease image (1 = no greasy tongue coating, 2 = mild greasy tongue coating, 3 = severe greasy tongue coating, 4 = curdy tongue coating).

The model detecting tongue indentation and spots was trained by manually using rectangular boxes to annotate abnormal-pixel positions. The tongue crack segmentation model was trained by manually marking the crack area (**Figure 4**).

The tongue features were statistically analyzed in two groups separately.

### High-throughput sequencing for tongue-coating microbiota

The tongue-coating microbiota of each participant were sampled using sterile swabs, disposable mouth mirrors, cryopreservation tubes, ice packs, and a portable incubator. The participant was informed previously not to brush teeth or eat after getting up in the morning. On the day of sampling, the participant should present no physical discomfort and did not drink, smoke, or chew sweets before sampling. Then, the participant rinsed his or her mouth with sterile water three times (10 mL each time) to remove food debris. Then, the researcher rolled forward a sterile swab along the middle of the participant’s tongue for three times (approximately 2-cm-long wiping action) and repeated this movement for two times. Afterward, the sterile swab was transformed into a cryopreservation tube and immediately transported to a -80°C freezer with a portable incubator filled with ice packs. This process was accomplished within an hour. Repeated freezing and thawing of samples were avoided. The samples were placed into a portable Styrofoam box with dry ice, then sent to the laboratories at Majorbio Bio-Pharm Technology Co. Ltd. (Shanghai, China) within a month, and preserved in a freezer at -80°C until nucleic acid extraction.

**Figure 5:**
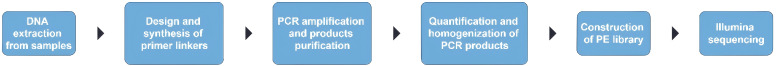
Sequencing and experiment workflow.

According to the manufacturer’s instructions, total DNA was extracted from tongue-coating microbiota samples using E.Z.N.A.^®^ Soil DNA Kit (Omega Bio-tek, Norcross, GA, USA). Agarose gel electrophoresis (1%) and a NanoDrop^®^ ND-2000 spectrophotometer (Thermo Scientific Inc., USA) were used to determine DNA quality and concentration. The hypervariable region V3–V4 of the bacterial 16S rRNA gene was amplified with primer pairs 338F (5′-ACTCCTACGGG-AGGCAGCAG-3′) and 806R (5′-GGACTACHVGGGTWTCTAAT-3′) by an ABI GeneAmp^®^ 9700 PCR thermocycler (ABI, CA, USA) (Liu et al., 2016). PCR amplification comprised denaturation at 95°C for 3 min, 27 cycles of denaturing at 95°C for 30 s, annealing at 55°C for 30 s, and extension at 72°C for 45 s, and single extension at 72°C for 10 min, and end at 10°C. The PCR reaction mixture was made by adding 4 μL of 5× Fast Pfu buffer, 2 μL of 2.5 mM dNTPs, 0.8 μL of primer (5 μM each), 0.4 μL of Fast Pfu polymerase, 10 ng of template DNA, and ddH_2_O to a final volume of 20 µL. Triplicate amplifications were performed on all samples. The PCR product was extracted from 2% agarose gel, purified using the AxyPrep DNA Gel Extraction Kit (Axygen Biosciences, Union City, CA, USA), and quantified using Quantus™ Fluorometer (Promega, USA). On an Illumina MiSeq PE300/NovaSeq PE250 platform (Illumina, San Diego, CA, USA), purified amplicons were pooled in equal amounts and paired-end sequenced (**Figure 5**).

**Figure 6:**
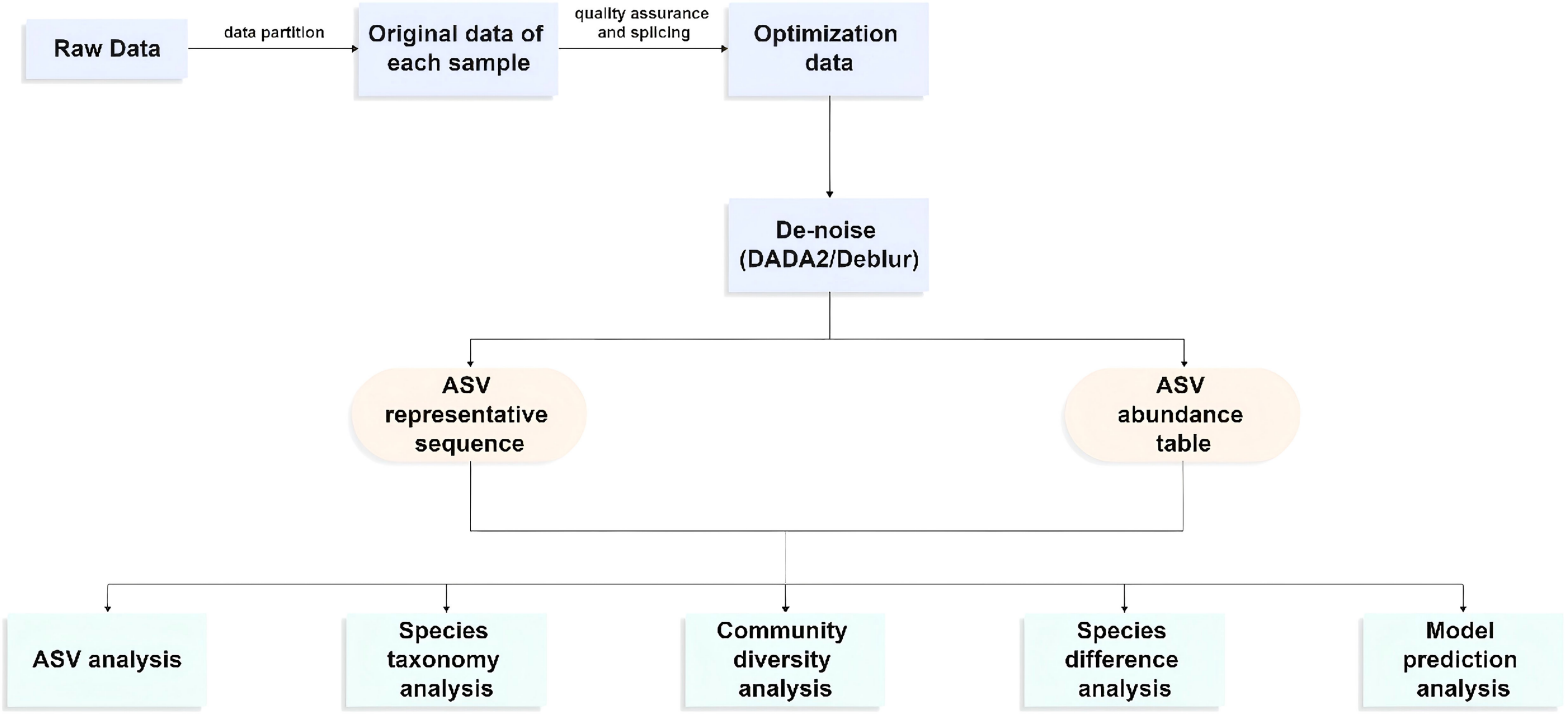
High-throughput sequencing workflow.

The resultant sequences were quality-filtered with Fastp (0.19.6) (Chen et al., 2018) and merged with FLASH (v1.2.11) (Magoc and Salzberg, 2011) after demultiplexing. Then, the high-quality sequences were denoised using DADA2 (Callahan et al., 2016) plugin in the Qiime2 (Bolyen et al., 2019) (version 2020.2) pipeline with default parameters to obtain a single-nucleotide (amplicon sequence variants) resolution based on the error profiles within the samples. DADA2-denoised sequences are usually called amplicon sequence variants (ASVs). The number of sequences from each sample was rarefied to 20,000 to minimize the impact of sequencing depth on alpha and beta diversity. With a contrast threshold set to 70%, the SILVA 16S rRNA database (v138) and the naive Bayesian classifier were used to assign taxonomic classifications to ASVs. The metagenomic function was predicted by using PICRUSt2 (Phylogenetic Investigation of Communities by Reconstruction of Unobserved States) (Douglas et al., 2020) based on the ASVs of representative sequences and abundances. A series of statistical or visual analyses were carried out, including ASV analysis, species taxonomy analysis, community diversity analysis, species difference analysis, and model prediction analysis (**Figure 6**).

### Statistical analysis

Clinical data were analyzed with R v4.2.1. The data were described as mean ± SD (standard deviation) if in normal distribution and otherwise as median with lower and upper quartiles. Student’s *t*-test and chi-square test were respectively used to assess differences between two groups of continuous and categorical variables. A *P*-value less than 0.05 was considered statistically significant.

The quantitative and visual analyses of tongue images were realized by both GMSX001, a tongue diagnosis instrument, and software Nahefa Cloud System V2.0. Nahefa Cloud System V2.0 is constructed based on techniques of deep learning object detection (Wang, 2023), deep learning image segmentation (Chen et al., 2018), and deep learning image classification (He, 2016).

The tongue-coating microbiota was subjected to bioinformatic analysis on the Majorbio Cloud platform. Mothur v1.30.2 and R v3.3.1 were used to analyze microbial diversity and calculate alpha diversity indices, including Sobs, Chao, Ace, Shannon, Simpson index, and Good’s coverage, based on ASV information (Schloss et al., 2009). The similarity among the microbial communities in different samples was determined by principal coordinate analysis (PCoA), principal component analysis (PCA), and non-metric multidimensional scaling analysis (NMDS) based on Bray–Curtis dissimilarity using Qiime software. Wilcoxon rank-sum test was used to analyze the difference in microbial community structure between groups. To identify the significantly abundant taxa (phylum to genera) of bacteria among the different groups, linear discriminant analysis (LDA) effect size (LEfSe) (Segata et al., 2011) was performed (LDA score > 3, *P* < 0.05).

Classical machine learning methods, including logistic regression (LR), support vector machine (SVM), and GBDT, were used for the construction of NPM-diagnosing models. Using stratified sampling method, healthy participants and NPM patients were randomly assigned in a 8:2 ratio to a training set (*n* = 160) or an internal test set (*n* = 40) to analyze the performances of different models. The predictive ability was illustrated based on area under the receiver operating characteristic curve (auROC) and decision curve analysis (DCA). The significance of each feature was inferred from the machine learning diagnosis model. Python v3.7 and scikit-learn v1.0.2 were used for modeling.

### Reporting guidelines

This study strictly adhered to the STROBE (Strengthening the Reporting of Observational Studies in Epidemiology) guidelines for reporting observational data collection and analysis. Complete checklists are provided in **Supplementary Table S1**.

## Results

### Participant characteristics

A total of 200 participants were included, including 100 NPM patients and 100 healthy people. The clinical characteristics of all participants are shown in **Table 1**. The average age (SD) was 30.42 (6.18) years in the healthy group and 32.34 (4.85) years in the NPM group. The NPM group showed higher BMI, WHR, and SP (*P* < 0.001) but no significant difference in DP.

**Table 1:** Clinical characteristics of included participants.

Items	Health	NPM	*P*-value
Number (person)	100 (50%)	100 (50%)	
Age (years)	30.42 (6.18)	32.34 (4.85)	0.015
Weight (kg)	55.40 [50, 62.25]	63.25 [55, 70]	<0.001
Height (cm)	1.62 [1.60, 1.67]	1.60 [1.58, 1.64]	0.011
BMI (kg/cm^2^)	20.96 [19.47, 23.42]	23.71 [21.64, 26.82]	<0.001
SP (mmHg)	109 [103, 117]	119.50 [110, 125.25]	<0.001
DP (mmHg)	71.41 (9.18)	73.87 (9.79)	0.068
Hip (cm)	92.50 [88, 99]	97.25 [93.38, 104.12]	<0.001
Waist (cm)	74 [68, 80]	84.50 [79, 91.62]	<0.001
WHR	0.79 (0.05)	0.86 (0.06)	<0.001

NPM, non-puerperal mastitis; BMI, body mass index; SP, systolic pressure; DP, diastolic pressure; WHR, waist–hip ratio.

### Tongue image features

Tongue image features included tongue color, tongue coating color, tongue coating thickness, tongue shape, tongue spot, tongue crack, and tongue indentation. To unify the format of data format, the tongue color and tongue coating color were recorded as standard chromaticity of the Lab color space specified by the International Commission on Illumination. Among them, the value of “L” represents the brightness of the pixel. The increased value of “A” means that the color changes from red to green. The increased value of “B” means that the color changes from yellow to blue (Billmeyer, 1983). These features were further subdivided according to their scores counted by Nahefa Cloud System V2.0.

The NPM group showed lighter tongue coating luminance and fewer tongue spots but yellower and thicker tongue coating than the healthy group (*P* < 0.05). No significant difference was observed in tongue color, tongue shape, tongue crack, and tongue indentation between groups (**Table 2**).

**Table 2:** Scores of tongue image features.

Items	Health (*N* = 100)	NPM (*N* = 100)	*P*-value
Tongue coating color-L	105.15 (14.09)	99.99 (13.37)	0.009
Tongue coating color-A	139.30 [137.85,140.32]	138.50 [137.50,140.46]	0.207
Tongue coating color-B	105.39 [103.25,107.41]	106.91 [103.40,110.95]	0.016
Tongue color-L	49.55 [46.34, 53.50]	49.43 [46.35, 52.14]	0.458
Tongue color-A	15.80 [13.66, 17.07]	14.42 [12.43, 16.92]	0.104
Tongue color-B	4.26 [3.43, 5.27]	3.65 [0.78, 6.13]	0.126
Tongue coating thickness	0.72 [0.56, 0.86]	0.83 [0.74, 0.89]	<0.001
Tongue shape	0.85 [0.83, 1.20]	0.84 [0.50, 0.87]	0.094
Tongue spot	35.50 [23.00, 54.25]	27.50 [12.00, 46.25]	0.003
Tongue crack	0.02 [0.00, 0.20]	0.00 [0.00, 0.09]	0.118
Tongue indentation	3.00 [1.00, 4.00]	3.00 [2.00, 4.00]	0.449

NPM, non-puerperal mastitis.

### Tongue-coating microbiota profiles

**Figure 7:**
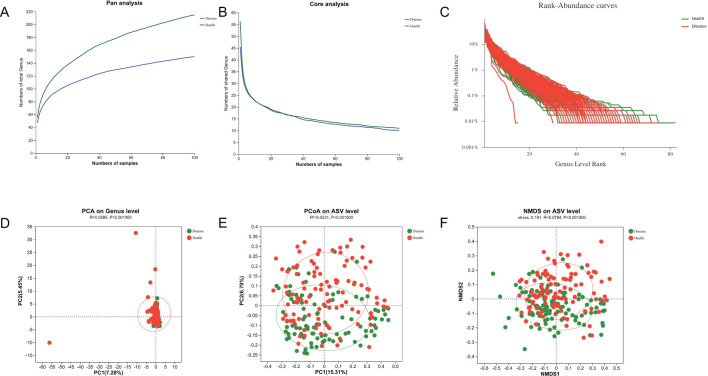
Microbial diversity analysis and β-diversities of tongue-coating microbiota. **(A, B)** Pan and core analysis of the NPM and health groups. **(C)** Rank–abundance curve of the NPM and health groups. **(D–F)** PCA, PCoA, and NMDS of binary Jaccard distance at the ASV/genus level. NPM, non-puerperal mastitis; PCA, principal component analysis; PCoA, principal coordinate analysis; NMDS, non-metric multidimensional scaling analysis; ASVs, amplicon sequence variants.

ASVs with 97% similarity were clustered into using Qiime2 (v2022.2) software and drawn according to the minimum number of sample sequences. A total of 10,889 ASVs were generated, with 1,276 and 8,519 ASVs in the NPM group and healthy group, respectively. The tongue-coating microbiota were further classified to one domain, one kingdom, 18 phyla, 29 classes, 73 orders, 129 families, 243 genera, and 542 species using the classify-sklearn (naïve Bayesian) algorithm. Pan/core analysis and dilution curve analysis suggested that the volume of sequencing data was large enough for further analysis (**Figures 7A–C**). Wilcoxon rank-sum test showed significant differences in community richness, diversity, and coverage indices between the two groups (all *P* < 0.05, **Table 3**). In addition, PCA, PCoA, and NMDS showed a significant difference in the distribution and dispersion of PC1/NMDS1 and PC2/MNDS2 axes as well as the aggregation area between the two groups (*P* = 0.001), indicating a significant difference in the microbial composition between the two groups (**Figures 7D–F**).

**Table 3:** α-Diversities in NPM and healthy groups.

Diversity index	NPM ( $\bar{x}\pm \text{s}$ )	Health ( $\bar{x}\pm \text{s}$ )	*P*-value	FDR
Sobs	134.62 ± 40.13	199.91 ± 74.78	<0.001	<0.001
Chao	137.27 ± 42.34	240.30 ± 111.00	<0.001	<0.001
Ace	137.46 ± 42.44	255.67 ± 131.68	<0.001	<0.001
Shannon	3.68 ± 0.32	3.55 ± 0.41	0.019	0.019
Simpson	0.05 ± 0.02	0.07 ± 0.04	0.006	0.007
Good’s coverage	1.00 ± 0.0005	1.00 ± 0.0003	<0.001	<0.001

NPM, non-puerperal mastitis; FDR, false discovery rate.

**Figure 8:**
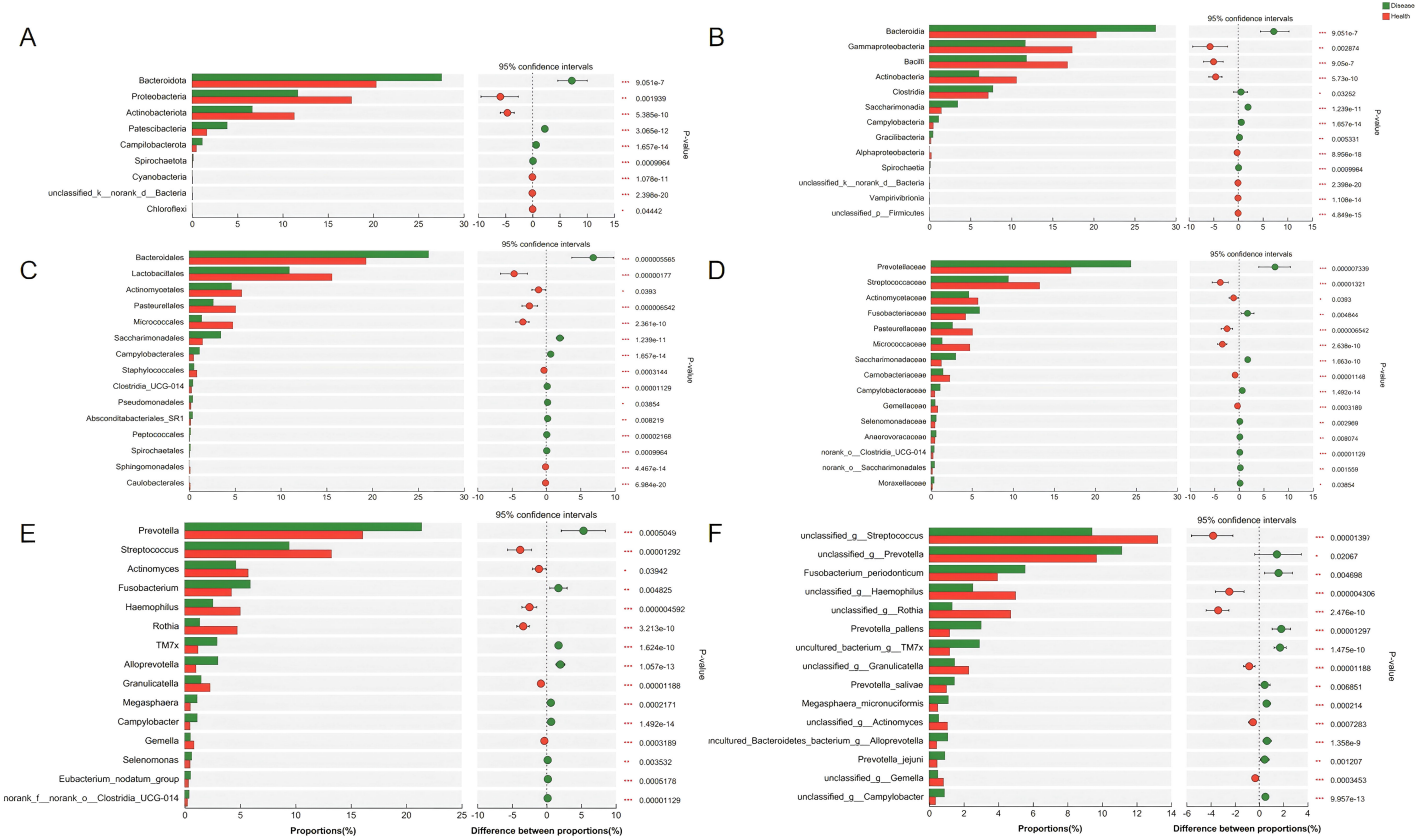
Bacterial taxa with differential abundances between the NPM and healthy groups. The bacterial taxa showed a significant difference at the phylum level **(A)**, class level **(B)**, order level **(C)**, family level **(D)**, genus level **(E)**, and species level **(F)**. NPM, non-puerperal mastitis.

Wilcoxon rank-sum test was further performed to evaluate the difference between NPM and healthy groups at each taxonomic level. Differences in microbiota species were assessed using LEfSe with the Kruskal–Wallis sum-rank test and LDA scores >3. The microbiota profile of the NPM group was significantly different from that of the healthy group, with differences in nine phyla, 13 classes, 15 orders, 15 families, 15 genera, and 15 species (all *P* < 0.05, **Figure 8**).

**Figure 9:**
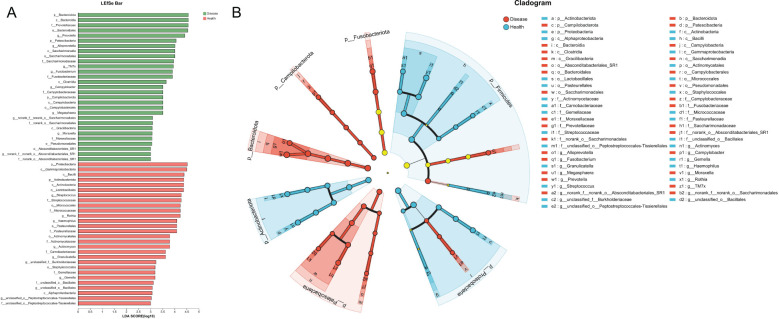
LEfSe analysis revealed the differential bacterial taxa between NPM and healthy groups. **(A)** Histogram plot showing the bacterial taxa with significantly different abundances between two groups (LDA > 3). **(B)** Cladogram showing the taxonomic tree of taxa with significantly different abundances between the two groups. LEfSe, linear discriminant analysis effect size; LDA, linear discriminant analysis; NPM, non-puerperal mastitis.

To find out the differential bacterial taxa, we performed LEfSe analysis, which confirmed that the NPM group showed increases in the phyla of *Bacteroidota*, *Patescibacteria*, classes of *Bacteroidia*, *Saccharimonadia*, *Clostridia*, *Campylobacteria*, *Gracilibacteria*, orders of *Bacteroidales*, *Campylobacterales*, *Pseudomonadales*, *Absconditabacteriales_SR1*, families of *Prevotellaceae*, *Saccharimonadaceae*, *Fusobacteriaceae*, *Campylobacteraceae*, *Moraxellaceae*, and genera of *Prevotella*, *Alloprevotella*, *TM7x*, *Fusobacterium*, *Campylobacter*, *Megasphaera*, *Moraxella* but decreases in the phyla of *Proteobacteria*, *Actinobacteriota*, classes of *Gammaproteobacteria*, *Bacilli*, *Actinobacteria*, *Alphaproteobacteria*, orders of *Lactobacillales*, *Micrococcales*, *Pasteurellales*, *Actinomycetales*, *Staphylococcales*, families of *Streptococcaceae*, *Micrococcaceae*, *Pasteurellaceae*, *Actinomycetaceae*, *Carnobacteriaceae*, *Gemellaceae*, and genera of *Streptococcus*, *Rothia*, *Haemophilus*, *Actinomyces*, *Granulicatella*, and *Gemella* compared to the healthy group (multi-group comparison strategy: all-against-all, LDA > 3, *P* < 0.05, **Figures 9A, B**).

### Performances of machine learning models in diagnosing NPM

**Figure 10:**
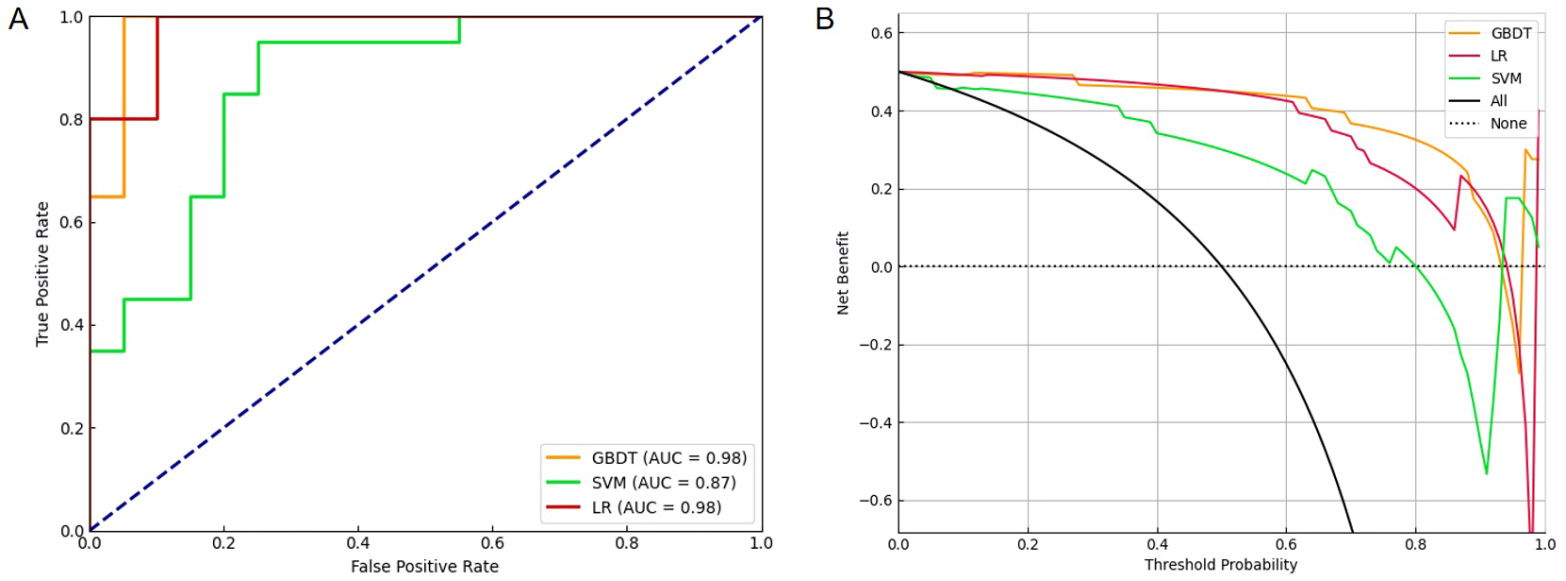
The ROC plot and decision curves revealed different performance among three machine learning models in diagnosing NPM. **(A)** The curves of true positive rate versus false positive rate among three machine learning models. **(B)** The clinical net benefit for each prediction model was calculated across a range of risk threshold probabilities. Decision curve analysis showing that GBDT had the highest net benefit in diagnosing NPM. ROC, receiver operating characteristic; NPM, non-puerperal mastitis; GBDT, gradient boosting decision tree.

To classify NPM patients, LR, SVM, and GBDT models were established using a combination of 44 features (including clinical characteristics, tongue images, and tongue-coating microbiota features). The features were selected through an integrated approach involving expert evaluation, literature review, and deep learning-based calculation. All models were operated in the same training and validation sets. The LR and GBDT models exhibited obvious advantages (auROC of 0.98), outperforming the SVM model (auROC of 0.87, **Table 4**; **Figure 10A**).

**Table 4:** Performance of the three machine learning models.

Model	Precision	Recall	Accuracy	Specificity	Sensitivity	AUROC
LR	0.95	0.95	0.95	0.90	1.00	0.98
SVM	0.80	0.80	0.80	0.75	0.85	0.87
GBDT	0.95	0.95	0.95	0.95	0.95	0.98

AUROC, area under the receiver operating characteristic curve; LR, logistic regression; SVM, support vector machine; GBDT, gradient boosting decision tree.

We next performed a DCA to evaluate the practicability of the three models. The SVM model provided the least net benefit, whereas the GBDT model provided the greatest gain. With threshold probabilities of risk ranging from 0.6 to 1.0, the gain from the GBDT model was particularly higher than those from the other two models, with added net incremental benefits across each threshold (**Figure 10B**).

To evaluate the performances of GBDT models with different characteristics, we incorporated seven types of features, including clinical characteristics, tongue image features, bacterial genera, bacterial families, bacterial species, and their different combinations (**Table 5**). The results showed that the models separately based on only clinical characteristics or a combination of clinical characteristics and tongue image features had similar performances (auROC of 0.90 to 0.92, model A–D). Models E, F and G, which are based on a combination of bacterial genera/species/families plus tongue image features and clinical characteristics, demonstrated a higher diagnostic accuracy, indicating that bacterial features could improve the accuracy of the GBDT model. Models E and G, separately based on a combination of clinical characteristics, tongue image features, and bacterial genera (model E) and families (model G), demonstrated the highest accuracy (0.95), specificity (0.95), and sensitivity (0.95). However, the performance of model F, which incorporated clinical characteristics, tongue image features, and bacterial species, was slightly worse. Based on the same amount of data, the diagnostic accuracy of the model decreased as the number of feature dimensions increased.

**Table 5:** Performance of the GBDT models based on different combinations of features.

Model	Features	Accuracy	Sensitivity	Specificity	AUROC
A	Clinical characteristics	0.85	0.80	0.90	0.90
B	Tongue image features	0.80	0.85	0.75	0.91
C	Bacterial species	0.85	0.85	0.85	0.90
D	Clinical characteristics + tongue image features	0.85	0.85	0.85	0.92
E	Clinical characteristics + tongue image features + bacterial genera	0.95	0.95	0.95	0.98
F	Clinical characteristics + tongue image features + bacterial species	0.88	0.80	0.95	0.98
G	Clinical characteristics + tongue image features + bacterial families	0.95	0.95	0.95	0.98

GBDT, gradient boosting decision tree; AUROC, area under the receiver operating characteristic curve.

**Figure 11:**
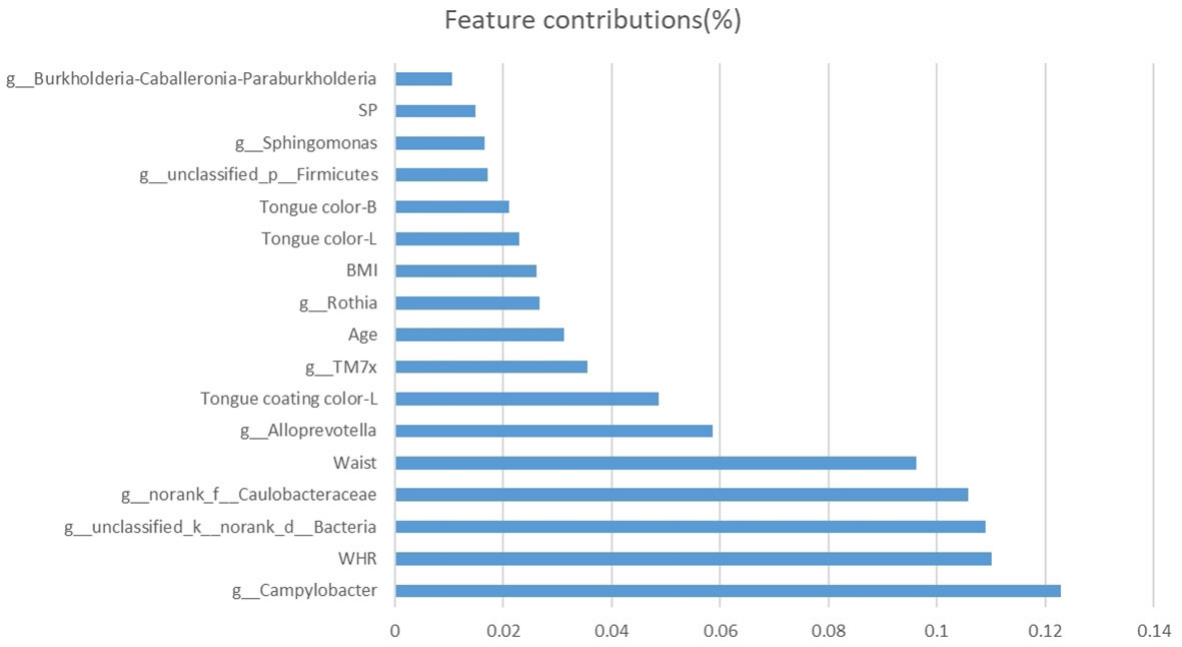
Bar plot of selected features and their contributions to the predictive ability of the NPM diagnosis model. NPM, non-puerperal mastitis.

The features with the closest associations with NPM risk in model E included *Campylobacter* (12%), WHR (11%), and waist circumstance (10%) followed by *Alloprevotella* (6%), tongue coating color-L (5%), *TM7x* (4%), age (3%), *Rothia* (3%), BMI (3%), tongue color-L (2%), and tongue color-B (2%, **Figure 11**).

## Discussion

NPM may arise from various etiological factors, ranging from infection to autoimmune disorders (Gopalakrishnan et al., 2015). The management of NPM is a thorny issue, and any misdiagnosis may lead to overtreatment, such as mastectomy (Bani-Hani et al., 2004). Our study is the first multi-modal analysis integrating tongue-coating microbiota and tongue image features from NPM patients and healthy people. We identified a cluster of microbial species and a list of tongue phenotypes associated with NPM. Besides that, combining the clinical, tongue image, and tongue-coating microbiota features, a GBDT model was established, showing a strong ability to screen out NPM. This model was non-invasive, simple, accurate, and highly suitable for large-scale NPM screening.

In our study, the mean WHR of NPM patients reached 0.86, indicating the association of central obesity with NPM risk. WHR, as the ratio of waist circumference to hip circumference, is effective to evaluate central obesity and predict the relationship between body fat distribution and the risk of various metabolic diseases. Even for a subject with a normal BMI, a higher WHR still increases the risk of premature death (Gazarova et al., 2022). According to the World Health Organization standard, the WHR of women should not exceed 0.85 (Nishida et al., 2010). Our results showed that WHR and waist circumstance were statistically different between the two groups. Studies have shown that obesity is a risk factor for NPM (Jiao et al., 2023). On the one hand, obesity may directly damage the immune function in the breast (Liu et al., 2017). Adipose tissue accumulates during development, but excessive accumulation may lead to hypoxia that increases the production of inflammatory factors and decreases that of anti-inflammatory factors, thus arousing inflammatory responses (Nishimura et al., 2008). Obesity also favors the development of mild chronic inflammation. Adipokines secreted by adipose tissue, such as visfatin, leptin, and acylated proteins, may disrupt neuroendocrine activities, thus inducing systemic inflammatory and immune responses (Fan et al., 2014; Liu et al., 2017). In addition, interferon-γ secreted by adipose tissue can directly act on estrogen receptors in the breast, thereby dysregulating estrogen and progesterone levels to evoke local immune responses and hypersensitivity (Brown, 2014).

In this study, the blood pressure was normal in both groups, while SP was slightly higher in the NPM group. Similarly, a research has shown that the incidence of hypertension is slightly higher in NPM patients compared with benign breast mass patients (OR, 2.221; 95% CI, 1.318–3.741; *P* = 0.003) (Shi et al., 2022). The association between hypertension and NPM needs to be further studied. Additionally, hypertension may increase the risk of breast cancer by 15% in women (Han et al., 2017), which may be explained by the fact that breast cancer and hypertension are driven by similar physiopathological pathways, such as chronic inflammation mediated by adipose tissue (Balkwill et al., 2005; Largent et al., 2006; Li et al., 2005).

Moreover, we innovatively combined the tongue image and tongue-coating microbiota features for diagnosing NPM. As a fundamental TCM methodology, tongue diagnosis is convenient and non-invasive for revealing the pathological changes in internal organs and warning diseases in the early stage (Han et al., 2016; Zhang and Zhang, 2015). Nowadays, tongue diagnosis is still being used for evaluating patients’ physical condition and disease stage (Huang et al., 2022; Jiang et al., 2021; Zhang et al., 2022). However, tongue diagnosis is always subjective, and its accuracy may be decided by many factors, such as brightness in the clinic. Machine learning technology can allow an objective evaluation about the tongue condition. Classical machine learning algorithms are powerful in analyzing structural data (Bini, 2018) and image features. In addition, these algorithms can also drill into sets of complex data (Dobrescu et al., 2020; Gao et al., 2020). In the present study, between-group differences were observed in the quantitative features of tongue images. The NPM group showed more yellower and thicker tongue coating than the healthy group. Tongue coating represents the accumulation of exfoliated mucosa cells, debris, and proliferation of microorganisms (Negrato and Tarzia, 2010). Medical studies have shown that the tongue coating is associated with the occurrence and prognosis of various diseases (Ali et al., 2021; Chen et al., 2024). According to TCM theory, a thick tongue coating is usually accompanied with phlegm-dampness and blood stasis (Anastasi et al., 2009; Kirschbaum, 2010), while a yellow tongue coating mirrors a hot interior condition (Jiang et al., 2012; Ye et al., 2016). These tongue features are also consistent with the pathology of NPM, which manifests a combination of heat, phlegm, and blood stasis.

Moreover, our research results showed that the NPM group had fewer tongue spots than the healthy group. Tongue spots originate in the fungiform papillae, which are enlarged and protrude to form awn-like spikes (Shahbake et al., 2005). In TCM, tongue spots indicate heat in the blood or excess heat in the internal organs (Wang et al., 2022). The number of tongue spots has been used for evaluating breast cancer (Lo et al., 2013). In this study, most of the patients had suffered a long-term NPM, which consumed too much Qi and blood to produce more tongue spots.

Compared with tongue diagnosis, the indices of tongue-coating microbiota are more objective for diagnosing NPM. We found significant differences at taxonomic levels between groups, including nine phyla, 13 classes, 15 orders, 15 families, 15 genera, and 15 species. Between-group differences were observed in the genera of *Actinomyces*, *Alloprevotella*, *Campylobacter*, *Fusobacterium*, *Gemella*, *Granulicatella*, *Haemophilus*, *Megasphaera*, *Moraxella*, *Prevotella*, *Rothia*, *Streptococcus*, and *TM7x*. Among them, *Campylobacter*, *Alloprevotella*, *TM7x*, and *Rothia* had the closest associations with NPM risk in the model.

Oral *Campylobacters*, also termed “emerging *Campylobacter* species”, can cause infections that may have been underreported (Costa and Iraola, 2019). Except for periodontitis, oral *Campylobacters* have been associated with extraoral infections, including gastroenteritis, irritable bowel disease, Barrett’s esophagus, gastroenteritis, appendicitis, Crohn’s disease, ulcerative colitis, empyema thoracis, cerebral microbleeds in stroke patients, peritonitis, and abscesses in the bone (Castano-Rodriguez et al., 2017; Kaakoush et al., 2015; Lam et al., 2011; Shiga et al., 2020; Warren et al., 2013). Apart from their own pathogenicity, microbiota and their metabolites enter into the systemic circulation, thereby inducing and aggravating inflammation (Gao et al., 2022). Pathogenic oral bacteria can induce the production of proinflammatory factors. IL-6, with a positive correlation with the abundance of *Alloprevotella* (Ye et al., 2024), is upregulated in both the serum and breast tissues of NPM patients (Liu et al., 2024). The upregulation of *Alloprevotella* expression in diarrheal irritable bowel syndrome suggests that *Alloprevotella* may exert pro-inflammatory effects (Tang et al., 2023).

*TM7x*, a member of phylum *Saccharibacteria* (*TM7*), is involved in host immune response (Domenech et al., 2013; He et al., 2015). *In vivo*, *TM7x* may directly repress the inflammatory response by forming a biofilm that hinders immune activation (Domenech et al., 2013). *TM7x* also inhibits the expression of TNF-α induced by XH001 in macrophages, thus achieving immune escape (He et al., 2015). The *Rothia* genus comprises Gram-positive aerobic bacteria commonly found in the oral and respiratory tracts. These bacteria have the potential to function as opportunistic pathogens, contributing to a range of infections, including endocarditis, pneumonia, peritonitis, and septicemia, particularly in individuals with compromised immune systems (Fatahi-Bafghi, 2021). Considering the close connections among *Campylobacters*, *Alloprevotella*, *TM7x*, *Rothia*, and inflammatory diseases, further research is needed to figure out whether these bacteria cause NPM as conditioned pathogens or by triggering the systemic immune response and producing pro-inflammatory factors.

Deep learning technology, due to its ability to process large amounts of data and identify relationships hidden deep inside biological data, has been utilized in the biochemical analysis of natural products, disease diagnosis, and treatment (Ma et al., 2023b; Seetharam et al., 2019). In this study, we constructed GBDT, SVM, and LR models for the diagnosis of NPM. GBDT algorithm, as a classic algorithm proposed by Friedman of Stanford University, has a strong ability in classification, regression, and feature selection. GBDT can illustrate the importance of features in the classification or regression model by calculating the average of the weight of features in each decision tree (JH, 2001). In the present study, the GBDT model exhibited the best performance. Both GBDT and LR models showed high precision and recall parameters and low false negative and positive rates in detecting NPM. However, the interactions among multimodal data—tongue images, microbiota, and clinical features—may exhibit highly complex nonlinear patterns. GBDT was more accurate to capture such intricate relationships, whereas the linear assumptions of LR might constrain diagnostic performance. Based on different combinations of clinical, tongue image, and tongue-coating microbiota features, all of the GBDT models were highly sensitive, accurate, and specific to NPM, suggesting their better discriminative and predictive performances.

However, our study still has some limitations. Concerning potential heterogeneity in NPM and inherent variability in tongue features, the group sample was relatively small, which may lead to the instability of models. Future work will involve external validation of the model in larger cohorts. The limited interpretability of GBDT remains a critical concern for clinical adoption. The association between the non-invasive biomarkers and NPM remains to be investigated by clinical and experimental studies. Tongue image features may vary with tongue position and other factors, which calls for standard operating procedures.

## Conclusion

The GBDT model incorporating clinical characteristics, “whole tongue” images, and tongue-coating microbiota may serve as a reliable tool for the early screening and diagnosis of NPM.

## Data Availability

The data presented in the study are deposited in the NCBI repository, accession number PRJNA1291263. Further inquiries can be directed to the corresponding authors.

## Supplementary material

Supplementary Table 1 STROBE checklist of items that should be included in the reports of observational studies.
