# Supplementary figures and images for: Diagnosis of non-puerperal mastitis based on “whole tongue” features: non-invasive biomarker mining and diagnostic model construction
Source: Front Cell Infect Microbiol. 2025 Jul 28;15:1602883. doi: 10.3389/fcimb.2025.1602883 (PMC12336138; doi:10.3389/fcimb.2025.1602883)

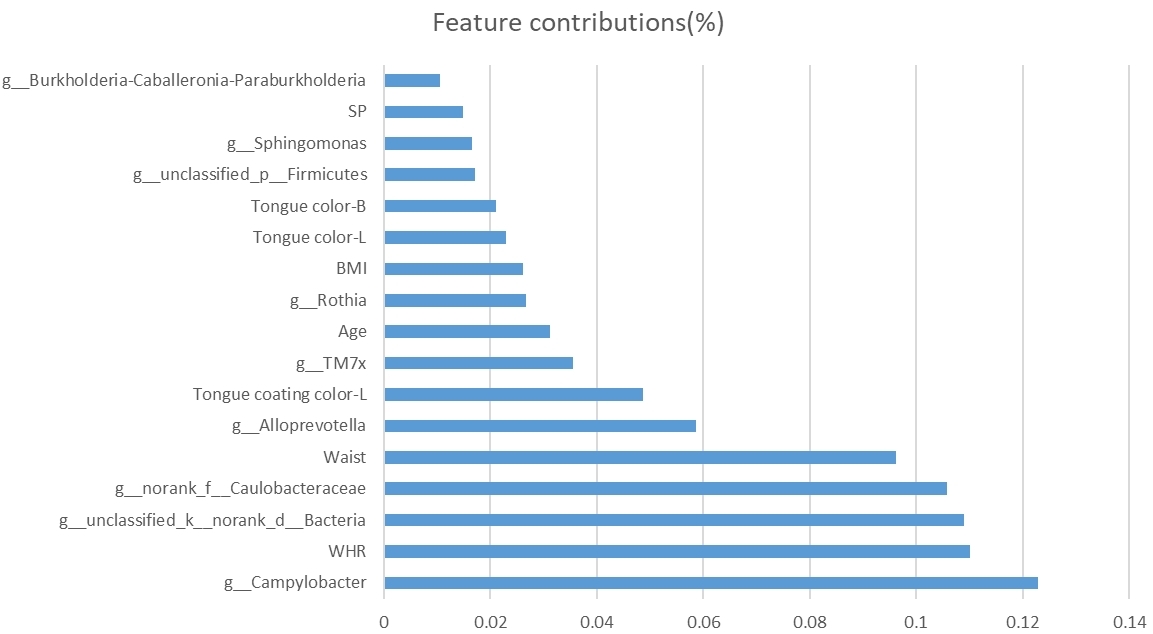

Supplement: Supplementary file 2 [file Image1.jpeg]
